# Anticoagulant treatment in COVID-19: a narrative review

**DOI:** 10.1007/s11239-020-02242-0

**Published:** 2020-08-18

**Authors:** Vincenzo Carfora, Giorgio Spiniello, Riccardo Ricciolino, Marco Di Mauro, Marco Giuseppe Migliaccio, Filiberto Fausto Mottola, Nicoletta Verde, Nicola Coppola, Vanvitelli COVID-19 group, Nicola Coppola, Caterina Sagnelli, Stefania De Pascalis, Maria Stanzione, Gianfranca Stornaiuolo, Angela Cascone, Salvatore Martini, Margherita Macera, Caterina Monari, Federica Calò, Andrea Bianco, Antonio Russo, Valeria Gentile, Clarissa Camaioni, Giulia De Angelis, Giulia Marino, Roberta Astorri, Ilario De Sio, Marco Niosi, Serena Borrelli, Benito Celia, Maria Ceparano, Salvatore Cirillo, Maria De Luca, Grazia Mazzeo, Giorgio Paoli, Maria Giovanna Russo, Vincenzo Carfora, Marco Di Mauro, Marco Giuseppe Migliaccio, Filiberto Fausto Mottola, Riccardo Ricciolino, Giorgio Spiniello, Nicoletta Verde

**Affiliations:** 1 Department of Translational Medical Sciences, University of Campania “Luigi Vanvitelli”, Naples, Italy; 2 Infectious Diseases Unit, Department of Mental Health and Public Medicine, University of Campania “Luigi Vanvitelli”, Via: L. Armanni 5, 80131 Naples, Italy

**Keywords:** COVID-19, SARS-CoV-2, Thrombosis, Anticoagulation

## Abstract

The actual Coronavirus Disease (COVID 19) pandemic is due to Severe acute respiratory syndrome coronavirus 2 (SARS-CoV-2), a member of the coronavirus family. Besides the respiratory involvement, COVID 19 patients frequently develop a pro-coagulative state caused by virus-induced endothelial dysfunction, cytokine storm and complement cascade hyperactivation. It is common to observe diffuse microvascular thrombi in multiple organs, mostly in pulmonary microvessels. Thrombotic risk seems to be directly related to disease severity and worsens patients’ prognosis. Therefore, the correct understanding of the mechanisms underlying COVID-19 induced prothrombotic state can lead to a thorough assessment of the possible management strategies. Hence, we review the pathogenesis and therapy of COVID 19-related thrombosis disease, focusing on the available evidence on the possible treatment strategies and proposing an algorithm for the anticoagulation strategy based on disease severity.

## Highlights

- SARS-CoV-2 induced complement hyperactivation, endothelial dysfunction and cytokine storm have a pro-thrombotic effect.
- COVID 19 patients develop a pro-coagulative state directly related to disease severity.
- In COVID 19 critical patients, thrombotic lesions in pulmunary microvessels have a prevalence twice higher than critical non-COVID 19 patients.
- Anticoagulant treatment is associated with lower mortality. Hence, we propose an algorithm for the anticoagulation strategy based on disease severity.

## Introduction

The actual Coronavirus Disease (COVID 19) pandemic is due to Severe acute respiratory syndrome coronavirus 2 (SARS-CoV-2), a member of the coronavirus family. It is a single strain RNA-virus that enters human cells through the binding between the viral structural spike (S) protein and the angiotensin-converting enzyme 2 (ACE2) receptor [1]. ACE 2 is mainly expressed on the surface of alveolar type II epithelial cells, cardiac myocytes and vascular endothelial cells (EC). Viral entry is facilitated by a type 2 transmembrane serine protease, TMPRSS2, via the S protein as well [2].

SARS-CoV-2 has rapidly spread worldwide, so far that in March 2020 the World Health Organization (WHO) has declared it a global pandemic and public health emergency. COVID-19 has a wide spectrum of possible clinical features, ranging from asymptomatic patients to severe pulmonary disease with multiorgan failure (MOF) [3]. Asymptomatic infection may be present in up to 85% of confirmed cases [4, 5].

There is a growing evidence of a procoagulant state in COVID 19 patients, with important prognostic implications. Lungs are the most affected organs and critically ill patients often show thrombotic lesions in pulmonary microvessels, with a prevalence twice higher than critical non-COVID 19 patients [6]. Therefore, the correct management of the anticoagulant therapy in COVID 19 patients has a fundamental role.

This narrative review will summarize the knowledge on the coagulative state in patients with COVID-19 and its therapeutic management. The article is addressed particularly to physicians having in care patients with COVID-19 in their clinical practice.

## Methods

A narrative review was performed using MEDLINE and Google Scholar from January 2020 up to 28th May 2020, in order to identify the coagulative state in patients with COVID-19. Last research was performed on 29th May 2020. We included the following search terms: “COVID-19” and “SARS-CoV-2” in combination with “Thrombosis” and “Anticoagulation”. The reference lists of all studies included were manually searched to identify any other study that might merit inclusion. We excluded articles in non-English-language.

## Biochemical and clinical manifestations of hyper-coagulable state in Covid 19

The autoptic evidence of diffuse microvascular thrombi in the lungs of patients died from COVID-19 support the theory of a hypercoagulable state. Carsana et al. [7] examined lungs tissues of 38 patients who died for COVID-19, with histologic evidence of platelet–fibrin thrombi in 33 out of 38 cases.

Tables 1 and 2 show the biochemical and clinical evidences, respectively, of hyper-coagulable status in COVID-19 (Tables 1, 2).

**Table 1:** Molecular evidence of hypercoagulable state in COVID-19

First author, year of publication (reference)	Evidence
Wang, 2020 [8]	In COVID-19-patients it is common to observe increased fibrinogen and D-Dimer levels
Chen, 2020 [9]	In COVID-19-patients it is common to observe variable levels of prothrombin time (PT), activated partial thromboplastin time (aPTT) and International standardized ratio (INR)
Qin, 2020 [15]	In COVID-19 the hyperinflammation mediated by IL-1, TNF-alfa and IL-6 leads to an increase of plasma concentrations of fibrinogen and plasminogen activator inhibitor-1 (PAI-1)
Campbell, 2020 [19]	In a murine model of MERS-CoV infection, increased concentrations of C5a and C5b-9 were found in sera and lung tissues. Blocking C5a with a murine antibody alleviated lung and spleen damage with decreased cytokine response and viral replication

**Table 2:** Biochemical and clinical evidence of hypercoagulable state in COVID-19

First author, year of publication (reference)	Evidence
Poissy, 2020 [6]	In 107 patients admitted in ICU for COVID 19 related pneumonia in 2020, pulmonary embolism (PE) had an unexpectedly high frequency (20.6%), being twice higher than what was observed in influenza patients admitted in ICU for respiratory failure in 2019
Carsana, 2019 [7]	Evidence of platelet–fibrin thrombi in lungs tissues of patients who died for COVID-19
Tang, 2020 [11]	183 consecutive COVID 19 patients have been enrolled and 15 of 21 non survivors had a ISTH-DIC score ≥ 5
Varga, 2020 [34]	Evidence of viral elements within endothelial cells in histological analyses,. These findings suggest that SARS-CoV-2 infection facilitates the induction of endothelitis in several organs as a direct consequence of viral involvement and of the host inflammatory response, leading to a prothrombotic state

In COVID-19-patients it is common to observe relative thrombocytopenia with increased fibrinogen and D-Dimer levels [8] and variable levels of prothrombin time (PT), activated partial thromboplastin time (aPTT) and International standardized ratio (INR) [9]. Moreover, there is evidence of direct correlation between D-Dimer levels and poor prognosis [10]. Tang et al. [11] enrolled 183 consecutive COVID-19 patients and performed routine coagulation tests [PT, aPTT, Fibrinogen, D-Dimer and fibrin degradation product (FDP)]. The 21 non-survivors (11.5%) showed significantly prolonged PT and aPTT and elevated D-Dimer, and 15 (71.4%) of these matched the grade of overt-DIC according to the International Society on Thrombosis and Hemostasis (ISTH) scoring system (*ISTH-DIC score ≥ 5*). These findings support the growing use of *ISTH-DIC score* as a prognostic score in COVID-19 patients. Thus, the hemostasis dysregulation leads to a prothrombotic state in COVID-19 patients and to microthrombosis formation in pulmonary small vessels of critical patients [12].

It is acknowledged that, regardless of etiology, critically ill patients have an increased risk of venous thromboembolism (VTE) [13] and this is particularly clear in severe COVID-19 patients. Poissy et al. [6] published a case series of 107 patients admitted in intensive care unit (ICU) for COVID 19 related pneumonia, showing that pulmonary embolism (PE) had an unexpectedly high frequency (20.6%), being twice higher than what was observed in influenza patients admitted in ICU for respiratory failure in 2019. Furthermore, in the reported PE cases there was a low number of associated deep vein thrombosis (DVT) suggesting that they had pulmonary thrombosis rather than pulmonary embolism from peripheral veins.

Because of the high PE incidence reported in critical COVID-19 patients, clinicians should suspect PE when there is hypoxemia disproportionate to respiratory disease, with or without acute unexplained right ventricular dysfunction, even in absence of typical DVT symptoms.

## Mechanisms of hyper-coagulable state in COVID-19

**Fig. 1:**
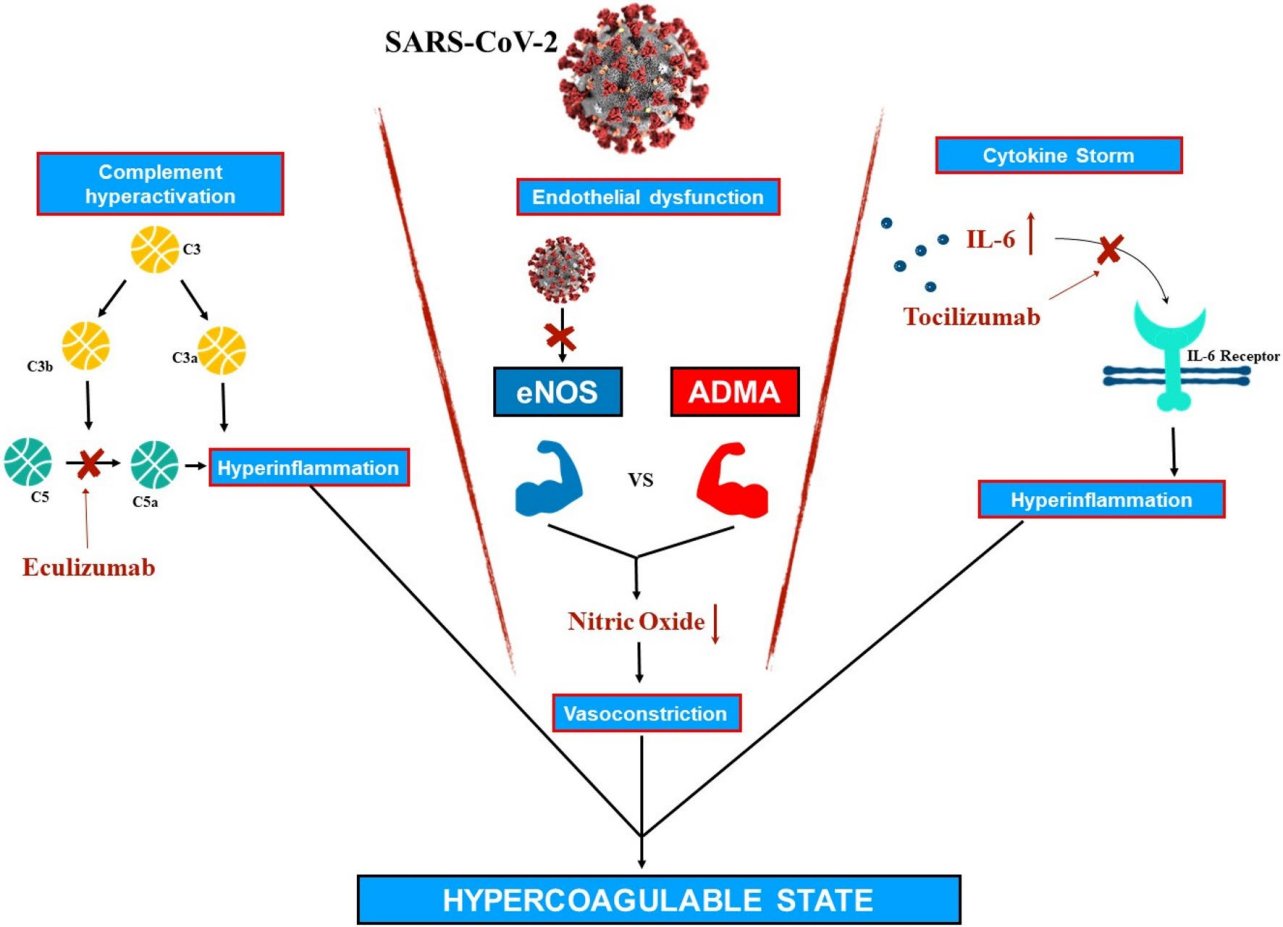
Hypercoagulable state pathogenesis in Covid 19 (*C3* complement component 3, *C5* complement component 5, *C3a* complement-activated product 3, *C5a* complement-activated product 5, *IL-6* interleukin 6, *eNOS* endothelial nitric oxide synthase, *ADMA* asymmetric dimethylarginine)

Figure 1 shows the possible mechanisms of the hyper-coagulable state in COVID-19 (Fig. 1).

COVID-19 patients can experience a hyper-inflammation phase, with a systemic response and a cytokine storm, that has a prothrombotic action [14]. In fact, as outlined by Qin et al. [15], in COVID-19 the hyperinflammation mediated by IL-1, tumor necrosis factor-alpha (TNF-α) and IL-6 leads to an increase of plasma concentrations of fibrinogen, lactate dehydrogenase (LDH), plasminogen activator inhibitor-1 (PAI-1) and neutrophil to lymphocytes ratio (NLR), mainly due to T CD4+ lymphocytes reduction. There is a close molecular interaction between inflammatory cytokines and coagulation. IL-6, IL-8, and TNF-α contribute to a pro-coagulant state promoting the activation of platelets, EC and the expression of tissue factor [16]. Furthermore, during inflammation there is a reduction in natural anticoagulants production such as antithrombin III, tissue factor inhibitor and Protein C, favoring a prothrombotic state [17]. Coagulation cascade can promote inflammation as well. In fact, thrombin is a major activator of protease-activated receptor 1 (PAR 1), a seven-transmembrane G-protein coupled receptor. PAR1 promotes the release of IL-1, IL-2, IL-6, IL-8, TNFα and increases the expression of adhesion molecules such as E- and P-selectin and ICAM-1 on the endothelial surface [18].

Another pathogenetic key-point in the pro-thrombotic effect of COVID-19 could be the pathological complement-activation, such as occurs in thrombotic micro-angiopathy (TMA) [19]. TMA can occur in different scenarios, as in Atypical Hemolytic Uremic Syndrome (aHUS), a rare disorder characterized by uncontrolled complement activation with hemolytic anemia, thrombocytopenia, and acute renal failure. In severe COVID-19, the reported elevated levels of LDH, d-dimer, and bilirubin, the mild thrombocytopenia and anaemia, the diffuse microvascular thrombi with renal and cardiac injury make the complement cascade hyperactivation a conceivable pathogenetic mechanism. Complement cascade activation converges in the activation of the C3 convertase that then cleaves C3 into C3a and C3b. C3b activates C5 convertase, which cleaves C5 into C5a and C5b. Thereafter, C5b forms a complex with other complement proteins, the C5b-9 membrane attack complex (MAC) that leads to cell lysis [20]. Complement cascade activation can lead to coagulation activation. In fact, C5a can increase tissue factor activity on EC, thus promoting coagulation cascade activation [21]. Furthermore, platelets have receptors for C3a that can promote their activation [22], while MAC adhesion on EC promotes the secretion of von Willebrand factor on their surface.

Thus, because of the prothrombotic role of the cytokine storm and of the probable complement cascade hyperactivation, humanized monoclonal antibodies (hMAbs) that target and inhibit these pathways are being used in COVID-19 clinical trials: Tocilizumab (hMAb that targets IL-6 receptor) and Eculizumab (hMAb that targets complement component 5 protein-C5).

As regards the endothelial disfunction, SARS-CoV-2 determines an endothelium damage with a pro-thrombotic effect. During the last few years, literature data demonstrated the association between endothelial dysfunction and thrombotic phenomena, since the endothelium plays an active role in the regulation of vascular tone and platelets activity, especially in case of hypoxia.

However, endothelium’s response to hypoxia varies in systemic and pulmonary circulation. In systemic circulation, hypoxia induces vasodilation by releasing nitric oxide (NO), while in pulmonary circulation, hypoxia induces vasoconstriction in order to reduce the perfusion of the unventilated lung areas [23].

SARS-CoV-2 binds the ACE-2 receptor, which is widely expressed on both alveolar type II epithelial cells and EC of the pulmonary microvessels. Lung epithelial cells act as oxygen concentration sensors, while EC modulate vascular tone through the release of vasoconstriction mediators like endothelin, superoxide radicals, arachidonic acid-derivatives, endoperoxides, thromboxane A2 and vasodilators like NO [produced by endothelial nitric oxide synthase 3 (NOS-3)]. The virus-related damage on EC leads to microvascular dysfunction, thus shifting the vascular equilibrium towards vasoconstriction with subsequent organ ischemia and inflammation, favoring a pro-coagulant state [24]. Vasoconstriction slows blood flow, thus favoring platelet activation and aggregation, while EC damage impairs their anticoagulant and fibrinolytic function, leading to platelet adhesion and coagulation cascade activation [25, 26]. One of the mechanisms involved is the impairment of the glycocalyx that covers EC surface of the vascular bed. EC glycocalyx plays an important role in nitric oxide-mediated vasodilation, and disruption of the glycocalyx results in thrombin generation and platelet adhesion [27]. Moreover, patients with cardiovascular disease and dyslipidemia have high levels of circulating asymmetric di-methyl-arginine (ADMA) [28], an analogue of L-arginine that inhibits NOS-3 activity [29], and this leads to lower NO levels; this explains why endothelial dysfunction and the pro-coagulant state are more severe in this cohort of patients.

According to these assumptions, statins [30] and ACE inhibitors (ACE-I) [31], because of their effect on reducing endothelial dysfunction, might find a rationale in the management of COVID-19 patients, as well as the reduction of low density lipoprotein (which reduces eNOS activity) [32] and the increased consumption of resveratrol (which induces eNOS activity and expression) [33].

Thus, thrombotic events observed in COVID-19 may be explained by the endothelial dysfunction, due to both direct virus-induced damage [34] and systemic inflammatory state, which has a pro-thrombotic effect [35]. This pro-coagulant state could explain the frequently observed pulmonary thrombi in COVID 19 patients that probably form in pulmonary vessels and are not the consequence of embolic propagation of peripheral venous thrombi [36].

## Management of hyper-coagulable in COVID-19

As stated before, cytokine storm and complement cascade hyperactivation probably play an important role in the hyper-coagulable state of COVID-19 patients and are the rationale for the use of anti-inflammatory drugs like hMAb that target and inhibit IL-6 pathway and complement cascade activation (tocilizumab and eculizumab, respectively). Xu et al. [37] treated 21 severe or critical COVID-19 patients with tocilizumab with routine therapy: fever ceased in all patients; 15 patients lowered their oxygen demand within 5 days after tocilizumab administration; C-reactive protein (CRP) levels decreased significantly in most patients; Computed tomography (CT) showed improvement of the lung opacities. Diurno et al. [38] published a case series on 4 severe COVID-19 patients treated with eculizumab: CPR values diminished in all patients, with evidence of lung lesions’ reduction on CT scans performed 48 h after Eculizumab administration in 3 out of 4 patients. Ongoing multicenter studies are further investigating Tocilizumab and Eculizumab use in COVID-19. NCT04317092 is a multicenter, open label, single arm study with primary endpoint of overall mortality reduction 1 month after registration and includes patients with SARS-CoV-2-induced interstitial pneumonia with respiratory insufficiency (O2sat ≤ 93% or PaO_2_/FiO_2_ ratio ≤ 300) treated with Tocilizumab. NCT04288713 (SOLID-C19) is a multicenter study investigating Eculizumab use in severe COVID-19 patients admitted in ICU with acute respiratory insufficiency, having as endpoints the reduction of mortality, time in ICU and time on a ventilator.

VTE risk assessment in non-COVID-19 inpatients relies on clinical validated scores like Caprini, Improve and Padua scores [39, 40]. However, it is not clear if these VTE risk scores should be used for COVID-19 patients or if these patients should routinely be treated with thrombus-prophylaxis because of their prothrombotic state. Tang et al. [41] retrospectively enrolled 449 severe COVID-19-patients and 99 (22.0%) patients received Low Molecular Weight Heparin (LMWH) treatment (4000–6000 IU q24h) for at least 7 days. D-dimer, PT and age were directly correlated with 28-day mortality, while low platelet count correlated with a worse outcome. LMWH treatment was associated with lower mortality in patients with *sepsis‐induced coagulopathy (SIC) score* ≥ 4 and in patients with D-dimer exceeding 3.0 μg/mL (6 times the upper limit of normal (ULN)). Lin et al. [42] suggest using LMWH at anticoagulant dose (100 IU/kg q12h) for at least 3–5 days in severe COVID-19 patients when D-dimer is higher than 4 times the ULN. Moreover, in support of routine use of LMWH, there is the evidence of the anti-inflammatory [43] and the protective action on endothelial function [44] exerted by LMWH.

Nowadays, clinicians agree on the use of LMWH in severe and critically ill COVID-19-patients, regardless of VTE risk scores. On the other hand, there are conflicting strategies on routine LMWH therapy in patients with mild and moderate COVID 19. For example, Zhai et al. [40] suggest that VTE risk-scores should be calculated in mild and moderate inpatients and routine thrombus-prophylaxis should be routinely used in severe and critically ill COVID-19-patients. Conversely, Cattaneo et al. [36] suggest a routine thrombi-prophylaxis with LMWH (4000–6000 IU q24h) in COVID-19 inpatients in absence of contraindications.

**Fig. 2:**
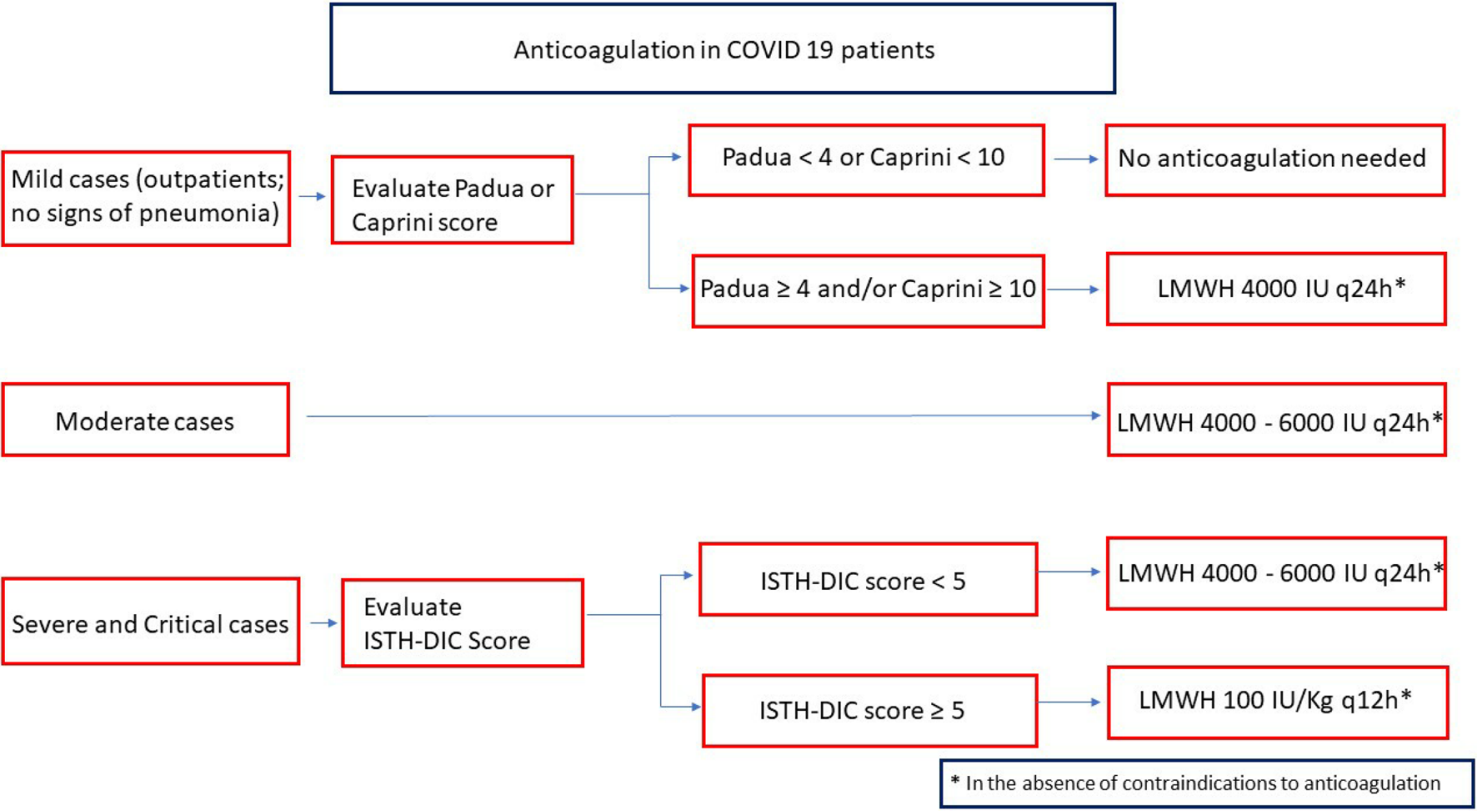
Proposed algorithm for anticoagulation strategy in COVID-19 patients (*LMWH* low molecular weight heparin, *q12h* every 12 h, *q24h* every 24 h, *ISTH-DIC score* International Society on Thrombosis and Hemostasis (ISTH) scoring system)

We propose the following algorithm for anticoagulation management in COVID 19 patients (Fig. 2). in *Mild cases*, Padua or Caprini scores should be evaluated; if Padua score is ≥ 4 and/or Caprini score is ≥ 10, LMWH (4000 IU q24h) should be started in the absence of contraindications.

In *Moderate cases,* LMWH (4000–6000 IU q24h) should be started, irrespective of VTE risk assessment scores, in the absence of contraindications. In *Severe and Critical cases:* ISTH-DIC score should be evaluated; in case of ISTH-DIC score < 5, LMWH (4000–6000 IU q24h) should be started, in the absence of contraindications; if the ISTH-DIC score is ≥ 5, we suggest to use full-dose LMWH (100 IU/Kg q12h), in the absence of contraindications.

## Conclusions

The whole scientific community is focusing on COVID-19 pandemic to face the hardest health emergency of the last years. The rapid and often sudden clinical deterioration of COVID-19-patients and the reported correlation between disease severity and D-Dimer levels often induce to search for PE in rapidly deteriorating patients. There is growing evidence that pulmonary thrombi directly form in situ, as a consequence of virus-induced inflammation and endothelial dysfunction, and this is particularly evident in patients with previous cardiovascular disease. Thus, the management of this hyper-coagulable status may have several treatment approaches.

Since SARS-CoV2 induces high IL-6 levels and complement cascade activation contributing to the prothrombotic state of COVID 19 patients, the use of anti-inflammatory drugs like corticosteroids and hMAb that target and inhibit IL-6 pathway and complement cascade activation (Tocilizumab and Eculizumab, respectively) may be useful [45, 46]. Likewise, drugs that reduce endothelial dysfunction, like Statins and ACE-I, could also play a role. Finally, LMWH is widely used in COVID-19 patients to prevent thrombi formation, but further studies are needed to assess the optimal anticoagulant regimen and to investigate other therapeutic strategies like platelet aggregation inhibitors.
